# As Ye Sow That Shall Ye Also Reap

**Published:** 1899-06

**Authors:** 


### EDITORIAL NOTES AND COMMENTS.

The Business office of THE JOURNAL-RECORD is 305, 306 Fitten Building.

The Editorial office is 318-319-320 The Prudential.

Address all Business communications to Dr. M. B. Hutchins, Mgr.

Make remittances payable to THE ATLANTA JOURNAL-RECORD OF MEDICINE.

On matters pertaining to the Editorial and Original Communications address Dr. Bernard Wolff, Atlanta.

Reprints of original articles will be furnished at cost price. Requests for the same should always be made *on the manuscript*.

We will present, post-paid, on request, to each contributor of an original article, twenty (20) marked copies of THE JOURNAL-RECORD containing such article.

### "AS YE SOW THAT SHALL YE ALSO REAP."

THE incineration of the negro assassin and rapist which occurred near this city was committed in the strict interpretation of the scriptural saying, "an eye for an eye and a tooth for a tooth." The punishment was inexpressibly horrible, but the crime for which it was meted out was sickening and revolting beyond portrayal.

It is not the function of a journal of this class to concern itself with the sociologic bearings of the episode. That belongs to the mentors and moralists of the pulpit and the secular press. But from the pathological standpoint from which we may view it, the negro was undoubtedly a moral idiot, a mere sketch of a man, in which the rough lines appeared without the delicate shadings of the completed individual. Swayed by reversively primitive emotions of revenge, hate and lust, he obeyed the imperious promptings of his dark and perverted nature. The fact that the negro was capable of committing rape, not once but several times successively, under circumstances and with environments that would have quenched the passion of the most lecherous Satyr that ever parodied man, suggests anthropological data sufficient to class him among sexual monsters.

Sadism is content to pinch and scratch and bite, but this cynical Caliban required the accompaniment of blood and brains to sate his seething wrath.

The mere accomplishment of the act was not sufficient; he must needs add to the degradation of his trembling and reluctant victim by forcing her to wash his genitals, and as a fitting climax to the orgie, with a final burst of erotic cynicism, he takes scissors and cuts off the hair from her pubes; then with a taunting laugh he leaves her with the ineffaceable stain of disgrace upon her and the scarcely less indelible imprint of syphilis.

Afterwards came the awful revenge by the people, and true to his instincts, his anesthetic stoicism broke down only when his scrotum was hacked off with a dull knife and the vessels and cords jerked out by the hand of an impatient avenger.

An editorial writer in the *Maryland Medical Journal* explains the cause of the frequency of rape by negroes upon white women in a way that bears conviction of truth to the minds of all who have examined the question from a physiologic standpoint. He says:

"The anatomical and physiological conditions of the African must be understood, his place in the anthropological scale realized, and his biological basis accepted as being unchangeable by man, before we shall be able to govern his natural uncontrollable sexual passions. When education and religious teachings change the biological basis of his color, it will also be able to change the physiological reason for his annual outbreak of sexual madness. Like all animal nature throughout the world, the African is especially sensitive to the changing seasons. The regular increase of crime against property in winter is only an indirect result, through the social and economic influences of temperature, but the increase of crimes of passion and indecent assaults during the month and years when the temperature commences to rise is the direct effect of temperature. The crime of rape is most numerous in May and June, and least so in November and December. Ignore the inherent and peculiar sexual organization of the African, and crimes against the trembling white women of the South will increase. Accept boldly, frankly and scientifically his ancestral traits, and control him accordingly, is the only rational, safe and moral treatment of the negro question." W.

